# Mindfulness, Interoception, and the Body

**DOI:** 10.3390/brainsci12060696

**Published:** 2022-05-27

**Authors:** Jennifer Todd, Jane E. Aspell

**Affiliations:** 1 School of Psychology and Sport Science, Anglia Ruskin University, Cambridge CB1 1PT, UK; jane.aspell@aru.ac.uk; 2 Centre for Psychological Medicine, Perdana University, Kuala Lumpur 50490, Malaysia

## 1. Introduction

In recent years, there has been a surge of interest in the topics of interoception and mindfulness from researchers, clinicians, and the general public alike (e.g., [1,2]). The rapid increase in research within both fields has led to difficulties in consistently defining and measuring the constructs. Regarding ‘mindfulness’, the term has roots in Buddhism, but has been adopted into Western contemporary culture to characterise a broad spectrum of practices, processes, and characteristics [3]. For parsimony, Kabat-Zinn [4] (p. 1481) suggests that mindfulness can be thought of as a “moment-to-moment, non-judgmental awareness, cultivated by paying attention in a specific way, that is, in the present moment, and as non-reactively, as non-judgmentally, and as openheartedly as possible”. In terms of defining interoception, researchers have recently acknowledged that it can be broadly defined as a collection of processes through which the nervous system senses, interprets, integrates and regulates information originating inside the body, providing a moment-by-moment mapping of the body’s internal environment [2,5].

There are clear theoretical parallels between the topics of interoception and mindfulness: traditional and contemporary methods for cultivating mindfulness are centred on the noticing of body sensations [3,6,7,8], and there are several interoceptive mechanisms that may impact mindfulness-based interventions, including interoceptive attention, interoceptive appraisal, and the use of interoceptive regulation strategies [9]. Reciprocally, interoceptive processing may be modulated through mindfulness-based training programmes via sustained attention to respiratory signals and bodily sensations such as pressure, movement and temperature, and cognitive and affective qualities of mindful attention to the internal body [8]. 

A growing body of research considers interoception and mindfulness in tandem (for interesting perspectives, see [3,6]). Within this body of literature, the associations between interoception and mindfulness appear to depend on the operationalisation of the two constructs [3]. For example, mindfulness-based training programmes have been found to enhance interoception when measured using self-report measures (e.g., [10,11,12,13]), and have also been associated with increased activation in the brain regions associated with interoceptive processing, such as the insular cortex [14,15,16,17]. However, there does not appear to be a consistent relationship between mindfulness and other components of interoception, such as interoceptive accuracy [18,19]. The interaction likely depends on the type of mindfulness practise employed and the timescale considered; for example, increases in interoceptive accuracy have been observed in training programmes utilising body scan mediations over a period of eight weeks [20] or over nine months of engaging in contemplative practices [21].

As a contribution to knowledge in this field, this Special Issue brings together six new articles (one review, and five original research studies) which consider the associations between multiple components of interoception and mindfulness-based interventions/training programmes, in clinical and non-clinical groups of adolescents and adults, from both Western and non-Western contexts. We summarise the findings from these studies below.

## 2. Clinical Research

Differences in interoceptive processing have been identified as an important component of several physical and mental health conditions [2,22,23], and modulations of interoceptive processing represent a promising avenue of novel therapeutic interventions for many mental health conditions [2,8,22,24]. Within this Special Issue, the first group of studies considered the associations between interoception and mindfulness-based interventions/training programmes (in particular, the use of mindfulness-based practices to modulate interoceptive processes) in adults with substance use disorders, depression, and suicidal ideation.

May and colleagues [25] provide new insights on the role of interoceptive dysfunctions in the development and maintenance of substance use disorders, and the potential utility of mindfulness-based interventions for the treatment of interoceptive symptoms in substance use disorders. In reviewing these themes, May and colleagues [25] suggest that substance use disorders may be typified by exaggerated responses in the insula and anterior cingulate cortices to drug-specific cues, but diminished behavioural performance and attenuated activity in the insula and anterior cingulate cortices during interoceptive tasks. Furthermore, May and colleagues [25] interrogate the limited research utilising mindfulness-based interventions in substance use disorders, which suggests that mindfulness-based interventions hold promise for the treatment of substance use disorders, but require further scrutiny to optimise the selection of approaches and doses, and to identify whether treatments are effective across different substance classes. 

In a different clinical context, Karanassios and colleagues [26] compared the effects of a standardised cognitive behavioural therapy (CBT) against CBT with an additional mindfulness-based stress reduction training programme on interoceptive abilities and depressive symptoms in adults with depression. Here, the authors found that participants with depression had a significant decrease in depression symptoms, and an increase in trait mindfulness and interoceptive abilities (heartbeat tracking task scores, task-related confidence ratings, and scores on the Body Awareness subscale of the Body Perception Questionnaire), following the four-week CBT programme. Contrary to the authors’ expectations, Karanassios and colleagues [26] found that CBT with additional mindfulness-based stress reduction training did not have any additional benefits for depressive symptoms, trait mindfulness, nor interoceptive abilities. 

Finally, Smith and colleagues [27] present two studies that build upon previous research from their group, which has identified interoceptive deficits as a correlate and possible risk factor for suicidal ideation and behaviour in various samples from the United States (e.g., [28,29,30]). Here, Smith and colleagues [27] found that their previous findings can be extended to a non-Western context, by identifying associations between interoceptive deficits and the current, past, and future likelihood of suicidal ideation in a sample of Indian adults. Furthermore, Smith and colleagues [27] found that Reconnecting to Internal Sensations and Experiences (RISE)—a four-session mindfulness-informed intervention designed to reduce suicidal ideation by improving interoception (first tested in US adults; [31])—had good levels of acceptability, and was associated with improvements in interoceptive dysfunction and reductions in suicidal ideation in a sample of Indian adults.

## 3. Non-Clinical Research

The three remaining studies in this Special Issue examined interactions between mindfulness and interoception in non-clinical samples. Yoga practises are considered as a type of body-focused mindfulness practise by many practitioners and authors (e.g., see [32]), and yoga has been found to increase body awareness and responsiveness (e.g., [33]). In the current Special Issue, Schillings and colleagues [34] report a study that investigated whether a single twenty-minute yoga session would have any effects on interoceptive accuracy and emotional experience. Compared to participants assigned to an inactive control or endurance session, the yoga session participants showed no significant changes in interoception (measured via the heartbeat tracking task), nor any changes in affect. The authors suggest that the negative findings are due to an insufficiently long engagement with the practise: it may be that a longer course of multiple yoga sessions would be needed to increase body awareness and to have consequent effects on interoceptive accuracy. They also acknowledge the limitation of using only a single measure of interoception, namely the heartbeat tracking task, the validity of which has been criticised heavily in the recent literature (e.g., [35]). Future research should employ additional/alternative measures of interoception, including self-report measures, and examine the effects of multiple and/or longer yoga sessions.

Self-report measures of body awareness (BA) were employed in the study by Pérez-Peña and colleagues [36], which examined the effects of mindfulness-based interventions on BA in a non-clinical sample. The authors employed the following multidimensional definition of body awareness: “the subjective, phenomenological aspect of proprioception and interoception that enters conscious awareness, which is modifiable by mental processes including attention, interpretation, appraisal, beliefs, memories, conditioning, attitudes and affect”, from Mehling and colleagues [37]. A number of authors have suggested that there are two main ‘attention/attitude styles’ towards bodily sensations. Maladaptive BA is avoidant, evaluative and linked to anxiety and other negative emotional states, whereas adaptive BA is accepting, non-judgmental and related to wellbeing [38]. There is some evidence that adaptive BA can be trained using mindfulness-based interventions (MBIs; [39]) but few studies have tested this with studies of sufficient power. The study by Pérez-Peña and colleagues [36] therefore investigated the effects of an 8-week MBI (MBCT) on BA and on measures of experiential avoidance, rumination, self-efficacy, and self-discrepancy. BA was measured using two questionnaires—the MAIA and the Functional Body Sensation Questionnaire—plus an indirect measure, the Modified Autobiographical Memory Test [40]. The latter measures the spontaneous tendency to report body sensations when recollecting emotional memories. The MBCT group showed significant increases in self-report measures of BA and reduced symptomatology, but showed no differences in the indirect BA measure. The effects of the MBI were, however, mediated by belief-related aspects of body awareness. The results support the idea that MBCT is a transdiagnostic intervention that can affect cognitive processes common to several psychological disorders. 

The final paper in the Special Issue [41] examined the neural effects of mindfulness training: in particular, how the insula—a key interoceptive brain area—might be modulated in healthy adolescents. Given that some studies have shown changes to interoception following mindfulness training, it is to be expected that changes to interoceptive brain regions also occur. Previous research has shown that adolescence is a period in which interoceptive processes are still developing (e.g., [42,43]), and it is also a time in which several psychopathologies can first emerge (e.g., [44]). Mindfulness training (MT) shows promise as an early intervention to help prevent mental health issues and increase wellbeing in adolescents [45], and it is possible it may exert its effects via changes to interoception. Yu and colleagues [41] sought to test whether MT causes changes in interoceptive brain regions in adolescents. They employed fMRI-based neurofeedback-augmented mindfulness training, and found the differential effects of neurofeedback runs on sub-regions of the insula: increases in the activation of the anterior insula and decreases in the posterior insula. Furthermore, anterior insula activity was negatively correlated with life satisfaction, and posterior insula activity was positively correlated with pain behaviour. The findings suggest that MT modulates insula activation in a way that may have clinical significance for adolescents, and thus more research in this area is warranted.

Altogether, the studies in this Special Issue point to important associations between interoception, mindfulness, and mental health. They reinforce previous findings that interoceptive abilities may be diminished in adults with substance use disorders, depression, and suicidal ideation, and point to the next steps for clinical contexts. In particular, the findings show that it may be possible to improve interoceptive abilities via short-term mindfulness-based interventions and cognitive behavioural therapy. In tandem with this, improvements in interoception may translate to symptom improvements in related psychopathology, including the conditions referenced throughout this series of articles. The studies on non-clinical samples are consistent with previous research showing that mindfulness interventions can also be effective in improving mental wellbeing in the non-clinical population. In sum, the articles indicate that mindfulness interventions may exert some of their effects via changes to interoceptive processing, in both clinical and non-clinical samples, but more research is needed to understand the underlying mechanisms. Not all mindfulness techniques invoke a focus on internal body sensations, and differences between approaches may explain their differing consequences on interoception and mental health (see [3]), as may individual differences in responsiveness. It is conceivable that, in the future, interoceptive/mindfulness interventions could be tailored not only to particular psychological disorders (e.g., the approach needed for anxiety would likely differ from that needed for depression), but also to suit particular individuals—akin to personalised medicine—depending on which intervention(s) an individual benefits most from, and can best engage with. Promising times lie ahead.
